# Editorial: Body temperature homeostasis: the biological thermostat

**DOI:** 10.3389/fnins.2024.1527270

**Published:** 2024-11-28

**Authors:** Yoshihisa Koyama, Sanae Hosomi, Kazuhisa Yoshiya

**Affiliations:** ^1^Department of Neuroscience and Cell Biology, Osaka University Graduate School of Medicine, Osaka, Japan; ^2^Addiction Research Unit, Osaka Psychiatric Research Center, Osaka Psychiatric Medical Center, Osaka, Japan; ^3^Department of Traumatology and Acute Critical Medicine, Graduate School of Medicine, Osaka University, Suita, Osaka, Japan; ^4^Department of Emergency and Critical Care Medicine, Kansai Medical University General Medical Center, Osaka, Japan

**Keywords:** thermostat, traumatic brain injury, epidural-related maternal fever, therapeutic hypothermia, heatstroke, raphe pallidus

In humans, as a homothermal species, body temperature is regulated around 37°C. Even at rest, organs like muscles, liver, brain, heart, and kidneys continuously generate heat. To maintain body temperature homeostasis, the body disperses excess heat to the environment (Hiraoka et al., 2014; Nakamura, 2011). Additionally, we adapt to environmental temperature shifts, with the nervous system acting as “***the biological*** ***thermostat***.”

Temperature information from the skin is conveyed to the cerebral cortex through the spinothalamic cortical tract, allowing us to consciously perceive temperature. It also reaches the preoptic area via the lateral parabrachial nucleus, influencing sympathetic nerves to shield the body from external temperature changes. This defense mechanism operates independently of conscious temperature perception (Yahiro et al., 2017). Known as the thermoregulatory center, the preoptic area is crucial for survival in variable environments, essential for homeothermic species like humans. Moreover, the preoptic area functions as a command center during infection by inducing fever (Nakamura, 2011). In such cases, cytokines from activated immune cells stimulate the production of prostaglandin E2 (PGE2) in endothelial cells, triggering fever in the preoptic area. Fever acts as a biological defense to hinder the proliferation of invading viruses and bacteria. Therefore, the thermoregulatory system centered in the preoptic area is vital for protecting the body from environmental fluctuations and infections.

However, the recent abnormal weather and the emergence of unknown viruses pose significant dangers to living organisms, as they can lead to body temperature fluctuations that overwhelm natural thermoregulatory systems. For instance, extreme heat caused by global warming and prolonged fevers result in heat stress, harming organisms (Kataoka et al., 2014). This heat stress can trigger neuroinflammation, which may impair memory and worsen neurodegenerative disease (Zhu et al., 2023). Additionally, cold stress, arising from extreme cold or conditions like cooler disease (a modern ailment), causes heightened sympathetic nervous system activity, leading to mental stress, and chronic pain (Miyamoto et al., 2017). Moreover, fever of unknown origin and psychogenic fever brought on by mental stress may disrupt the biological thermostat, potentially causing its system to break down. To overcome these challenges, we need to learn more about ***the*** ***biological thermostat***. This Research Topic addresses this Research Topic with articles from thermoregulation and thermomanagement that cover clinical settings and diseases.

Verduzco-Mendoza et al. investigated the effects of traumatic brain injury (TBI) extending to the striatum on peripheral and core temperatures, as well as on monoamine levels in rats. They found that TBI with striatal injury led to increased temperatures and sustained motor deficits, along with elevated levels of noradrenaline and serotonin in the brain cortex and hypothalamus. In contrast, rats with TBI alone showed no temperature changes and quickly recovered motor function. These findings suggest that TBI with striatal hemorrhagic extension significantly impacts thermoregulatory function and monoamine levels compared to TBI alone. These changes following TBI may be linked to localized damage at the injury site and alterations in autonomic thermoregulatory processes.

The pathophysiology of epidural-related maternal fever (ERMF) is not fully understood, with two proposed hypotheses: inflammation from local anesthetics and thermoregulatory changes associated with epidural anesthesia. The mini-review by Kinishi et al. focuses on recent advances in understanding how the hypothalamus regulates body temperature and inflammation in ERMF. The authors highlight the significance of the interleukin-1 receptor antagonist in the sterile inflammatory fever pathway and the impact of hormones on temperature regulation during childbirth, which is crucial to understanding the pathophysiology of ERMF. This review incorporates the most recent information and suggests new avenues for research, offering a fresh perspective on ERMF and its management within the broader scope of autonomic neuroscience.

Ito et al. discuss temperature management in therapeutic hypothermia. Currently, therapeutic hypothermia is used in various fields, including organ transplantation, and cardiac anesthesia. In particular, it is often used in the treatment of central nervous system diseases because of its good prognosis. This review provides the latest knowledge in the field of temperature management in brain injury and cardiac arrest by closely examining clinical studies, mainly RCTs and guidelines on temperature management.

Yoneda et al. discuss heatstroke, a health problem unique to modern society. Record-breaking heatwaves caused by global warming are increasing the number of heatstroke patients every year, causing severe damage to body tissues, especially the central nervous system. This review provides a detailed description of the clinical characteristics, pathophysiology, and treatment of heatstroke, providing information for developing effective treatment strategies for brain damage caused by heatstroke and for promoting understanding of the long-term health effects of heatstroke.

Raphe Pallidus (RPa) is a brainstem nucleus containing sympathetic premotor neurons that regulate thermogenesis and cardiovascular function. Hitrec et al. expand our understanding of how orexin signaling affects behavior and autonomic functions in the RPa. They demonstrate that these effects vary with temperature, suggesting that orexin cannot stimulate an autonomic response without a pre-existing thermogenic drive. Additionally, the findings indicated that the RPa plays a role in promoting wakefulness.

Since we are continually exposed to both external and internal factors that can disrupt the body's temperature homeostasis, the thermoregulatory mechanism plays a crucial role in an organism's defense. This Research Topic's focus on the interaction between the nervous system that controls thermoregulation and the elements that interfere with temperature stability, gathers together the latest findings on the subject. We hope that these insights will aid in uncovering new neuroregulatory mechanisms of bodily homeostasis related to thermoregulation and in developing treatments for intractable febrile diseases.
